# Psychiatric hospitalization as a site of stigma reduction: a prospective cohort study from a specialty mood disorders unit

**DOI:** 10.3389/fpsyt.2026.1904948

**Published:** 2026-08-05

**Authors:** Edwina Awuku-Aboagye, Karen L. Swartz, Sarah C. Collica

**Affiliations:** Department of Psychiatry and Behavioral Sciences, Johns Hopkins School of Medicine, Baltimore, MD, United States

**Keywords:** inpatient psychiatry, length of stay, mood disorders, patient attitudes, prospective cohort, psychiatric hospitalization, stigma, therapeutic milieu

## Abstract

Although stigma associated with psychiatric hospitalization may discourage patients from seeking care, little is known about how patients’ views change after experiencing inpatient treatment. We examined admission-to-discharge changes in attitudes toward psychiatric hospitalization among adults admitted to the Johns Hopkins Mood Disorders Units, with particular attention to stigma worry and whether those changes were related to clinical improvement. In this prospective cohort study, 122 participants were enrolled, and 64 had paired admission-discharge attitude data. Participants completed a study-specific Attitudes Questionnaire at both time points. The primary stigma item asked whether participants worried what others would think if they learned of their psychiatric admission. Paired changes were evaluated using Wilcoxon signed-ranks tests, with exploratory analyses using Kruskal–Wallis and Spearman rank correlation tests. Eight of ten paired attitude items improved significantly. Agreement with stigma worry decreased from 65.6% at admission to 39.1% at discharge, a 26.5 percentage-point reduction; mean stigma worry decreased from 3.70 to 2.92, *z* = 4.41, *p* < 0.001. Participants with high baseline stigma worry had the greatest reductions, Kruskal–Wallis χ²(2) = 16.37, *p* = 0.0003; mean change was −1.21 in the high-stigma group versus +0.18 in the low-stigma group, *post-hoc p* = 0.0001. Longer length of stay was associated with greater stigma reduction, ρ = −0.290, *p* = 0.020. Stigma change was not significantly associated with PHQ-9, GAD-7, or PMQ-9 improvement. These findings suggest that inpatient treatment may reduce hospitalization-related stigma through processes not explained by symptom improvement alone.

## 1 Introduction

Mental disorders are among the leading contributors to years lived with disability worldwide, with depressive and anxiety disorders accounting for substantial global burden (1). In the United States, national survey data show that mental illness remains highly prevalent among adults, underscoring the need for effective systems of care across outpatient, crisis, and inpatient settings (2). Inpatient psychiatric services remain an important component of the acute mental health care continuum, providing evaluation, stabilization, and treatment for patients whose needs exceed what outpatient or crisis services can safely address (3). However, the landscape of inpatient psychiatric care in the United States has contracted substantially: psychiatric bed capacity has declined, and the average length of stay for many general psychiatric admissions is considerably shorter than the length of stay observed in specialty inpatient programs (3). These structural pressures make it increasingly important to understand what patients gain from hospitalization beyond symptom relief.

A central but underexamined dimension of the hospitalization experience is stigma. Stigma refers to a social process in which individuals who possess a devalued attribute are labeled, stereotyped, and subjected to status loss and discrimination (4). In the context of mental illness, several forms of stigma exist. Public stigma refers to the negative attitudes and discriminatory behaviors that members of the general population direct toward people with mental illness. Self-stigma occurs when individuals with mental illness become aware of these public attitudes, agree with them, and apply them to themselves (4). Internalized stigma refers to the incorporation of these stigmatizing beliefs into one’s self-concept, resulting in feelings of shame, reduced self-worth, and social withdrawal (5). Mental health-related stigma is well established as a barrier to care-seeking, treatment engagement, disclosure, and social reintegration (6). However, stigma specifically directed at psychiatric hospitalization, the feared social consequences of being identified as someone who required inpatient psychiatric care, is less well characterized. Within modified labeling theory, persons who enter psychiatric treatment may apply culturally learned beliefs about devaluation and discrimination to themselves, shaping self-concept, social behavior, and expectations of rejection (7). This hospitalization-specific anticipated stigma may exceed the discrimination patients actually encounter. Whether direct inpatient experience revises these anticipated beliefs, and whether hospitalization can itself be destigmatizing, remains almost entirely unexamined in prospective research.

Direct inpatient experience may also challenge stigma through contact, normalization, and therapeutic engagement. Contact-based approaches are a core component of stigma reduction efforts, and national guidance has emphasized the importance of social contact in reducing discrimination toward people with mental and substance use disorders (4, 8). In inpatient settings, therapeutic milieus expose patients to peers experiencing similar symptoms, structured psychoeducation, multidisciplinary treatment, and repeated interactions with psychiatric staff. Contemporary reviews of therapeutic milieu interventions and inpatient psychiatric patient experience highlight the importance of environment, staff relationships, peer interaction, and perceived safety in shaping care experiences and outcomes (9, 10).

Most stigma research has focused on public stigma, self-stigma, or broad attitudes toward mental illness. Instruments such as the Internalized Stigma of Mental Illness scale assess subjective stigma experiences among persons with psychiatric disorders but do not specifically measure worry about being known to have required psychiatric hospitalization (5). This distinction matters clinically. Hospitalization-related stigma concerns a specific care event that may influence willingness to seek inpatient care, engage with treatment, disclose admission to others, and integrate the experience into recovery after discharge.

The Johns Hopkins Mood Disorders Units (MDUs), specialty inpatient services that provide an intensive therapeutic milieu, offer a naturalistic setting in which to examine these questions. Drawing on an ongoing prospective cohort study of adults admitted to these units, we examined whether attitudes toward psychiatric hospitalization and stigma worry in particular change from admission to discharge and whether greater exposure to inpatient treatment is associated with larger change.

## 2 Methods

### 2.1 Study design and setting

The parent study, Longitudinal Outcomes Study of the Mood Disorders Units on Meyer 4 is an ongoing prospective cohort study of adults admitted to the Johns Hopkins Mood Disorders Units on Meyer 4. The parent study was designed to establish an MDU cohort and data registry to characterize patients treated on the units, evaluate short- and longer term treatment outcomes, identify prognostic factors associated with those outcomes, and assess patient attitudes toward psychiatric hospitalization. The study has two linked components: an electronic health record-based MDU data registry projected in Databricks on the Precision Medicine Analytics Platform, and a consented prospective cohort in which newly admitted patients complete study-related REDCap questionnaires at admission, discharge, and 6-month post-discharge follow-up. This brief report focuses specifically on the parent study aim related to patient attitudes toward hospitalization, with emphasis on stigma, and presents interim admission-to-discharge findings from data collected between November 2024 and April 2026.

The setting for recruitment comprised two specialty inpatient units: the Young Adult Mood Disorders Unit (six beds), which primarily serves transitional age patients between 18 and 26 years old, and the Adult Mood Disorders Unit (nine beds), which primarily serves patients aged 26 and older. These services provide multidisciplinary inpatient treatment for persons with mood disorders and related psychiatric conditions. Both units deliver an intensive therapeutic milieu comprising structured group programming, peer interaction, psychoeducation, and multidisciplinary treatment. Length of stay in this cohort was longer than is typical of contemporary short-stay psychiatric hospitalization models, permitting examination of whether greater exposure to inpatient treatment was associated with larger attitude change. At the time of the interim data extract, 122 participants had been enrolled. The primary analytic sample included 64 participants with paired admission and discharge Attitudes Questionnaire data.

### 2.2 Participants

Eligible participants were adults aged 18 years or older admitted to the Mood Disorders Units with an ICD-10 mood disorder diagnosis or related qualifying condition, including major depressive disorder, bipolar I disorder, bipolar II disorder, persistent depressive disorder, posttraumatic stress disorder, perinatal mood disorder, or mood disorder due to another medical condition. Participants were required to have English literacy, decision-making capacity, and ability to provide written informed consent.

The full descriptive sample included all enrolled participants in the interim dataset. The primary attitude-change analysis included participants who completed the Attitudes Questionnaire at both admission and discharge. Clinical symptom analyses were limited to participants with paired admission and discharge data for the relevant symptom scale.

### 2.3 Instruments and measures

The Attitudes Questionnaire was a study-specific instrument developed for the parent study. It included 11 items at admission and 13 items at discharge; each rated on a 5-point Likert scale from 1 = completely disagree to 5 = completely agree. Items 2–11 used parallel wording at admission and discharge, with tense changes only, and were treated as paired items (e.g., Item 2: “Other patients had a positive impact on my care”). Item 1 was excluded from paired analysis because the admission version assessed current apprehension about being admitted, whereas the discharge version assessed anticipated future apprehension relative to the current hospitalization. These represented related but non-equivalent constructs. Discharge Items 12 and 13, assessing willingness to return to the program and willingness to recommend the program, were analyzed descriptively only.

The primary stigma item was Item 8: “I am worried about what people will think of me if they find out I was admitted to a psychiatric unit.” Higher scores indicated greater stigma worry; therefore, a decrease from admission to discharge represented improvement. For descriptive presentation, 5-point Likert responses were collapsed into three categories: disagree, neutral, and agree. Collapsing was used for descriptive presentation only, given low cell frequencies in individual response categories, most notably the neutral category (*n* = 5 at admission); all inferential analyses used the full 5-point scale. Each attitude questionnaire item was analyzed individually; no composite total score was computed, so internal consistency statistics are not applicable to this instrument.

Clinical symptom measures included the Patient Health Questionnaire-9 (PHQ-9) for depressive symptoms, the Generalized Anxiety Disorder-7 (GAD-7) for anxiety symptoms, and the Patient Mania Questionnaire-9 (PMQ-9) for manic symptoms. The PHQ-9 is a validated 9-item depression severity measure (11). A sample item is “Little interest or pleasure in doing things,” with responses ranging from 0 (*not at all*) to 3 (*nearly every day*). Clinically meaningful PHQ-9 improvement was defined as a ≥5-point reduction based on prior treatment-monitoring work (12). The GAD-7 is a validated 7-item anxiety scale. A sample item is, “Feeling nervous, anxious, or on edge,” rated on the same 0–3 response scale. Clinically meaningful GAD-7 improvement was defined as a ≥4-point reduction (14). The PMQ-9 is a validated 9-item patient-reported measure of manic symptoms (15). A sample item is, “Over the past week, how often have you … had racing thoughts,” with responses ranging from 0 (*not at all*) to 3 (*nearly every day*). Because no validated minimal clinically important difference threshold is specified for the PMQ-9, a 50% reduction in the PMQ-9 was reported exploratorily for this measure. In the present sample, internal consistency at admission was Cronbach’s α = 0.88 for the PHQ-9, 0.92 for the GAD-7, and 0.83 for the PMQ-9, consistent with the values reported in the original validation studies (α = 0.89, 0.92, and 0.88, respectively) (11, 13, 15).

### 2.4 Statistical analysis

Descriptive statistics were used to summarize demographic, clinical, and hospitalization characteristics. Categorical variables were reported as frequencies and percentages. Continuous variables were reported as means with standard deviations, medians, interquartile ranges, and observed ranges where appropriate.

The primary analysis used Wilcoxon signed-ranks tests to evaluate admission-to-discharge change in paired Attitudes Questionnaire Items 2–11. This approach was selected because Likert responses were ordinal, bounded, and non-normally distributed, and because the design required within-participant paired comparisons. For each item, we reported mean admission and discharge scores, mean change, percent agreement at both timepoints, percentage-point change in agreement, and *p*-values.

Exploratory analyses evaluated potential predictors and correlates of stigma change. Baseline stigma group was defined using admission Item 8 as low, neutral, or high. Kruskal–Wallis tests compared stigma change across baseline stigma groups, with *post-hoc* Mann–Whitney *U* tests for pairwise comparisons. Spearman correlations assessed associations between length of stay and changes in stigma and other attitude items. Additional exploratory analyses assessed differences by prior inpatient history, gender group, diagnosis, age group, and MDU service. Associations between clinical symptom improvement and stigma change were assessed using Spearman correlations and Mann–Whitney *U* tests comparing stigma change by clinical improvement status. Sensitivity analyses compared paired and non-paired participants. Analyses were conducted using Stata 15.0.

### 2.5 Ethics

The study was approved by the Johns Hopkins School of Medicine Institutional Review Board (IRB00422772). All participants provided written informed consent before participation.

## 3 Results

### 3.1 Sample characteristics

Of the 122 enrolled participants, 64 had paired admission and discharge attitude data and formed the primary analytic sample. Paired data were unavailable for 58 participants, primarily because of participant non-response to the discharge questionnaire. Participant characteristics are shown in **Table 1**. The sample had a mean age of 29.5 years (*SD* = 11.7), with 47.5% aged 18–25 years. Women comprised 49.2%; 23.8% identified as men, 7.4% as non-binary, 4.9% as transgender, and 1.6% as other. Gender data were missing for 13.1%. The Young Adult Mood Disorders Unit included 67 participants (54.9%), and the Adult Mood Disorders Unit included 55 participants (45.1%).

**Table 1:** Participant characteristics (*N* = 122).

Characteristic	*n* (%) or mean (*SD*)
Demographics	
Age, years, mean (*SD*); range	29.5 (11.7); 18–72
18–25 years	58 (47.5)
>25 years	64 (52.5)
Gender	
Women	60 (49.2)
Men	29 (23.8)
Non-binary	9 (7.4)
Transgender	6 (4.9)
Other	2 (1.6)
Missing	16 (13.1)
Relationship status	
Single/never married	60 (49.2)
Married	23 (18.9)
Significant relationship	18 (14.8)
Education	
Some college or less	61 (50.0)
Bachelor’s degree	23 (18.9)
Master’s or higher	21 (17.2)
Employment at admission	
Full-time	41 (33.6)
Part-time	17 (13.9)
Unemployed/none	28 (23.0)
Household income	
< $50,000/year	35 (28.7)
$50,000–$149,000/year	32 (26.2)
≥ $150,000/year	20 (16.4)
Diagnoses, multiple permitted	
Major depressive disorder	69 (56.6)
Generalized anxiety disorder	51 (41.8)
Any bipolar disorder, I or II	36 (29.5)
PTSD	27 (22.1)
OCD	21 (17.2)
Mean diagnoses per participant	1.87 (1.35)
Hospitalization history and setting	
Prior psychiatric hospitalization	55 (45.1)
First-ever psychiatric admission	49 (40.2)
Young Adult Mood Disorders Unit	67 (54.9)
Adult Mood Disorders Unit	55 (45.1)
Length of stay, days	19.7 (13.5); median 16, IQR 10–25

Values are *n* (%) unless otherwise stated. PTSD, posttraumatic stress disorder; OCD, obsessive-compulsive disorder.

Major depressive disorder was the most common diagnosis (56.6%), followed by generalized anxiety disorder (41.8%), any bipolar disorder (29.5%), posttraumatic stress disorder (22.1%), and obsessive-compulsive disorder (17.2%). Participants had a mean of 1.87 diagnoses (*SD* = 1.35). Prior inpatient psychiatric hospitalization was reported by 45.1%; 40.2% were experiencing their first-ever psychiatric admission; and 14.7% did not indicate whether this was a first or repeat hospitalization. Most participants arrived via the emergency department; the remainder were either admitted from home (25.4%), transferred from a medical unit (5.7%), or transferred from another psychiatric unit (1.6%), and for 14.8%, admission source was unreported. The mean length of stay was 19.7 days (*SD* = 13.5; median = 16; IQR = 10–25; range: 3–77).

At admission, PHQ-9 data were available for 75 participants, with a mean score of 15.6 (*SD* = 7.3). GAD-7 data were available for 78 participants, with a mean score of 11.6 (*SD* = 6.4). These values indicated substantial depressive and anxiety symptom burden at admission.

### 3.2 Primary stigma item

Among the 64 participants with paired attitude data, stigma worry decreased significantly from admission to discharge. Agreement with Item 8 decreased from 65.6% at admission to 39.1% at discharge, a 26.5 percentage-point reduction. Mean stigma worry decreased from 3.70 to 2.92, Wilcoxon *z* = 4.41, p < 0.001.

**Figure 1:**
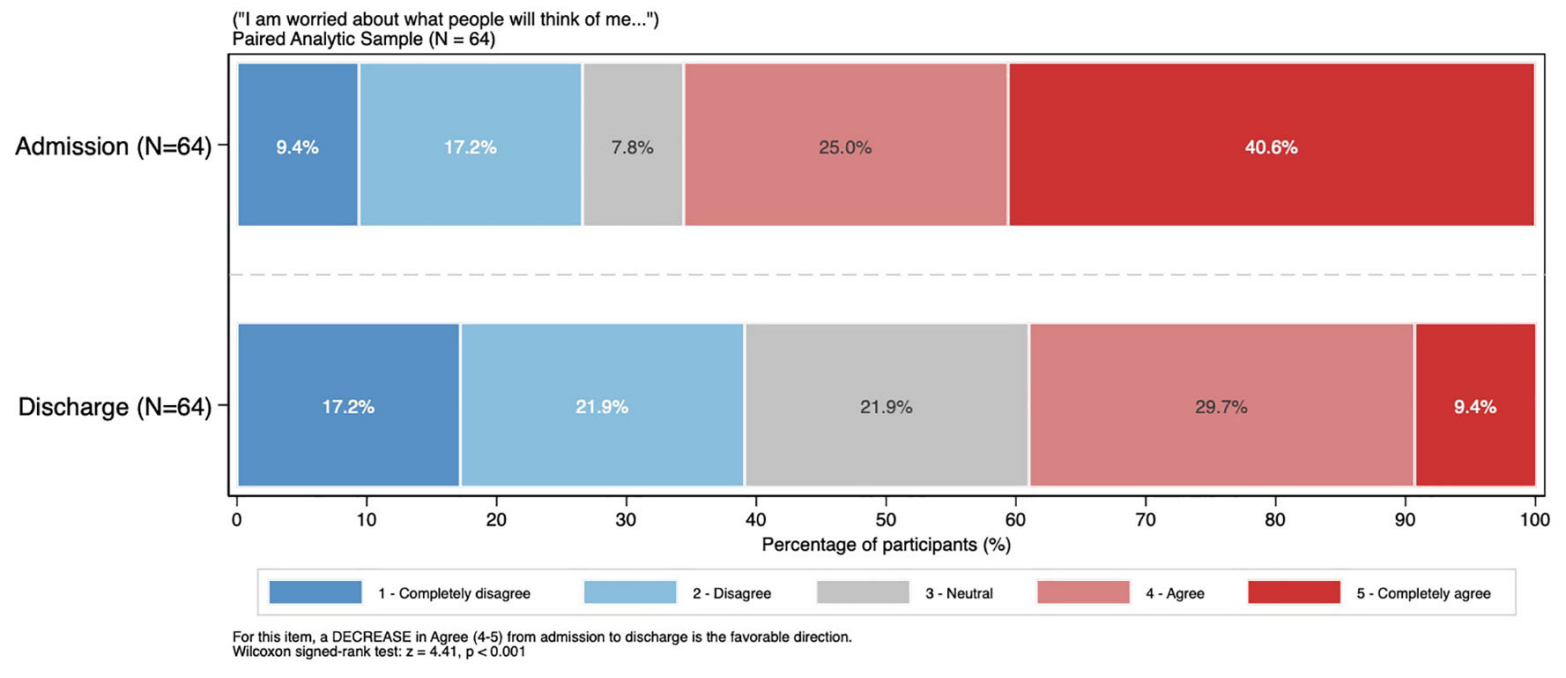
Distribution of responses to Item 8 (“I am worried about what people will think of me if they find out I was admitted to a psychiatric unit”) at admission and discharge in the paired analytic sample (*N* = 64). Response categories: 1 = Completely disagree to 5 = Completely agree. For this item, a decrease in endorsement of categories 4–5 from admission to discharge represents a favorable outcome (reduction in stigma worry). Wilcoxon signed-rank test: z = 4.41, *p* < 0.001.

The distribution shifted in the favorable direction. At admission, 26.6% disagreed, 7.8% were neutral, and 65.6% agreed that they worried what people would think if others learned of the psychiatric admission. At discharge, 39.1% disagreed, 21.9% were neutral, and 39.1% agreed. The change was concentrated at the extreme of the scale: the proportion selecting “completely agree” fell from 40.6% at admission to 9.4% at discharge, and the proportion selecting “completely disagree” rose from 9.4% to 17.2%, while endorsement of “agree” was similar at both timepoints (25.0% and 29.7%). **Figure 1** illustrates this shift.

### 3.3 All paired attitude items

Eight of ten paired attitude items improved significantly from admission to discharge. The largest mean changes were observed for patients’ understanding of the treatment for their psychiatric disorder, which increased from 3.48 to 4.33, Δ = +0.85, *p* < 0.001, and perceived positive therapeutic impact of other patients on the unit, which increased from 3.38 to 4.08, Δ = +0.70, *p* < 0.001. Knowledge of their psychiatric diagnosis increased from 3.95 to 4.58, Δ = +0.63, *p* < 0.001. Perceived adequacy of family or loved-one contact increased from 4.06 to 4.55, Δ = +0.49, *p* = 0.002, and perceived benefit of admission to overall health increased from 4.14 to 4.50, Δ = +0.36, *p* = 0.003.

Two paired items did not reach statistical significance. Perceived benefit of groups on the unit increased from 3.52 to 3.88, *p* = 0.111. Feeling included in care decisions by the treatment team increased from 4.25 to 4.38, *p* = 0.090, but agreement was already high at admission, suggesting a ceiling effect. Full paired item results are shown in **Table 2**.

**Table 2:** Paired attitude item results: Admission versus discharge *(N* = 64).

Item	Adm mean (*SD*)	Dis mean (*SD*)	Mean change	Adm % agree	Dis % agree	Δ % agree	*p*-value
2. Other patients had a positive impact on my care	3.38 (1.05)	4.08 (0.95)	+0.70	40.6	73.4	+32.8	**< 0.001**
3. Group therapy was beneficial to my treatment	3.52 (1.38)	3.88 (1.10)	+0.36	59.4	70.3	+10.9	0.111
4. Staff treated me with respect and dignity	4.08 (0.97)	4.45 (0.92)	+0.37	73.4	90.6	+17.2	**0.005**
5. I was included in treatment decisions	4.25 (0.86)	4.38 (0.96)	+0.13	85.9	87.5	+1.6	0.090
6. I had enough contact with family and friends	4.06 (1.09)	4.55 (0.82)	+0.49	76.6	90.6	+14.0	**0.002**
7. Admission was positive for my job or studies	3.45 (1.21)	3.84 (1.13)	+0.39	48.4	67.2	+18.8	**0.012**
8. I worried about what people would think of me *†*	3.70 (1.38)	2.92 (1.22)	−0.78	65.6	39.1	−26.5	**< 0.001**
9. Being admitted was beneficial to my health	4.14 (0.85)	4.50 (0.63)	+0.36	79.7	92.2	+12.5	**0.003**
10. I know my psychiatric diagnosis	3.95 (1.05)	4.58 (0.65)	+0.63	65.6	90.6	+25.0	**< 0.001**
11. I understand my prescribed treatment	3.48 (1.11)	4.33 (0.83)	+0.85	53.1	85.9	+32.8	**< 0.001**

†Item 8 is the primary stigma item. Higher scores indicate greater stigma worry; a decrease is the favorable direction for this item only.

Item 5 had a ceiling effect at admission, with 85.9% already agreeing.

Item 2 had *n* = 63 and Item 7 had *n* = 62; all other paired item analyses had *n* = 64.

Agree = responses 4–5 on a 5-point Likert scale from 1 = completely disagree to 5 = completely agree.

Wilcoxon signed-rank tests were used.

Values in bold are statistically significant.

Non-paired descriptive items also indicated favorable attitudes. At admission, 58.7% agreed that they were apprehensive about being admitted. At discharge, 61.5% agreed that they would be less apprehensive if they needed future psychiatric admission. At discharge, 80.0% agreed that they would return to the units if indicated, and 81.5% agreed that they would recommend the units to peers in need.

### 3.4 Clinical scale changes

Clinical symptom analyses were conducted using PHQ-9, GAD-7, and PMQ-9. Among participants with paired PHQ-9 data (*n* = 35), mean PHQ-9 score decreased from 16.8 to 8.9, *p* < 0.001. A clinically meaningful PHQ-9 improvement of at least 5 points was achieved by 24 participants (68.6%), 19 participants (54.3%) achieved at least 50% reduction, and 8 participants (22.9%) reached remission, defined as discharge PHQ-9 ≤4. The proportion of participants in the mild-or-below PHQ-9 range increased from 22.7% at admission to 50.0% at discharge.

Among participants with paired GAD-7 data (*n* = 39), mean GAD-7 score decreased from 11.6 to 7.9, *p* = 0.0004. A clinically meaningful GAD-7 improvement of at least 4 points was achieved by 17 participants (43.6%), 14 participants (35.9%) achieved at least a 50% reduction; and 17 participants (43.6%) reached remission, defined as a discharge GAD-7 ≤ 4. The proportion in the mild-or-below GAD-7 range increased from 39.7% at admission to 64.3% at discharge.

Among participants with paired PMQ-9 data (*n* = 32), mean PMQ-9 score decreased from 8.8 to 4.9, *p* < 0.001. A 50% reduction was observed in 18 participants (56.3%). These clinical findings provide context for attitude changes but were not the primary outcome of this report.

### 3.5 Predictors of stigma change

**Figure 2:**
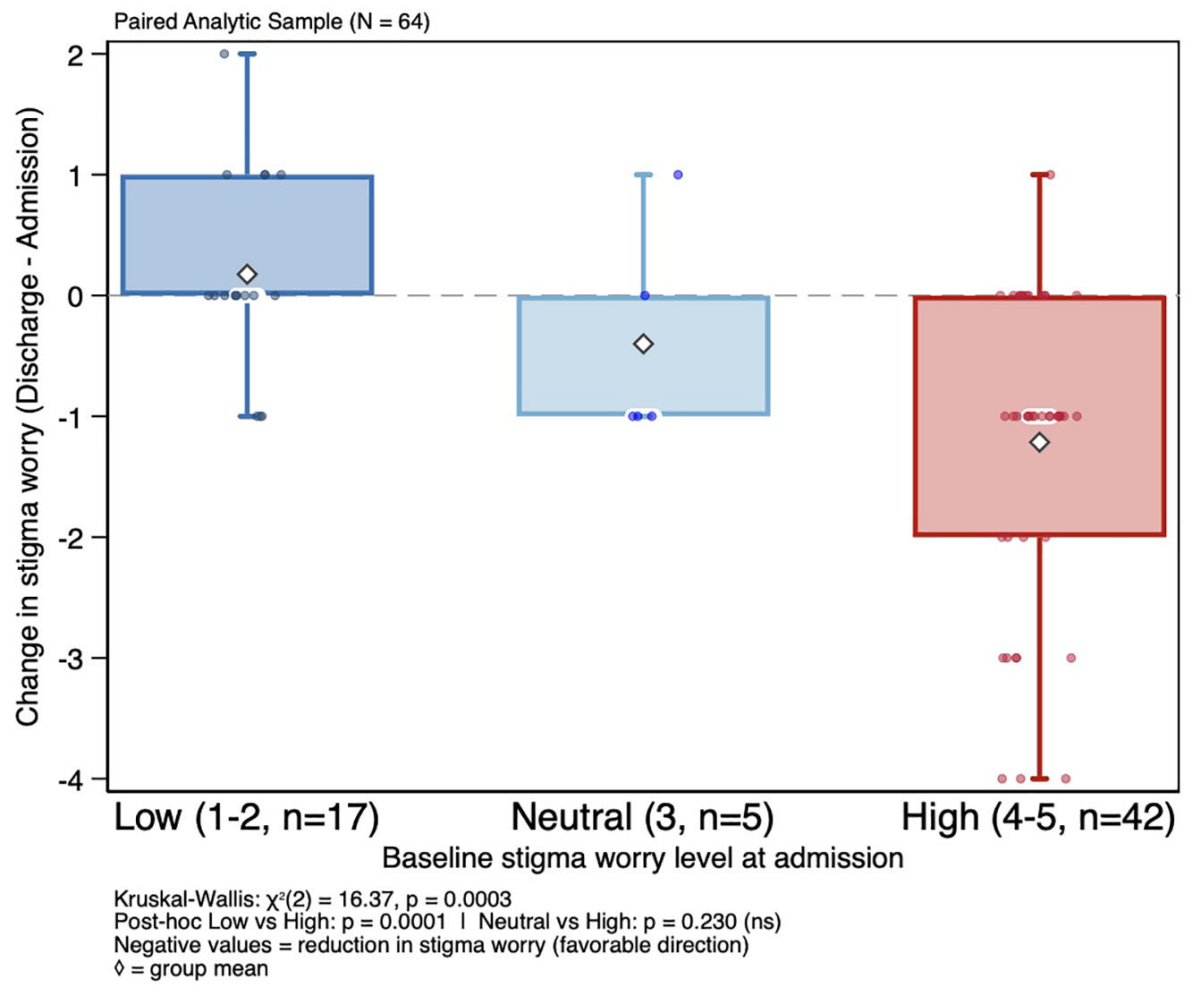
Change in stigma worry (Item 8 score: discharge minus admission) by baseline stigma level at admission, in the paired analytic sample (*N* = 64). Box plots show median (horizontal line), interquartile range (box), and range (whiskers); diamonds indicate group means; individual data points are overlaid with horizontal jitter. Low group: score 1–2 at admission (*n* = 17); Neutral: score 3 (*n* = 5); High: score 4–5 (*n* = 42). Negative values indicate reduction in stigma worry (favorable direction). Kruskal-Wallis χ²(2) = 16.37, *p* = 0.0003; *post-hoc* Low vs. High: *p* = 0.0001.

Baseline stigma level strongly predicted the degree of stigma change at discharge. Participants with high baseline stigma worry showed the largest reductions in stigma worry. Mean stigma change was +0.18 (*SD* = 0.81) among participants in the low baseline stigma group, −0.40 (*SD* = 0.89) in the neutral group, and −1.21 (*SD* = 1.26) in the high baseline stigma group. The difference was statistically significant, Kruskal-Wallis χ²(2) = 16.37, *p* = 0.0003. *Post-hoc* testing showed that the low and high baseline stigma groups differed significantly, *z* = 3.98, p = 0.0001. **Figure 2** displays stigma change by baseline stigma group.

Length of stay was associated with greater stigma reduction, with longer hospitalization correlated with larger decreases in stigma worry (Spearman ρ = −0.290, *p* = 0.020). This association appeared specific to stigma change, as length of stay was not significantly associated with changes in the other paired attitude items (all |ρ| < 0.21 and all *p* > 0.09).

Prior inpatient history was not significantly associated with admission stigma worry or stigma change. Admission stigma worry was 3.25 among participants with repeat admissions and 3.63 among first-time admission participants, *p* = 0.220. Stigma change was −0.55 among repeat-admission participants and −1.00 among first-time admission participants, *p* = 0.383. In exploratory subgroup analyses conducted for selected conditions, stigma change did not differ significantly by gender group, MDD versus bipolar diagnosis, or comorbid anxiety status.

Age group was not significantly associated with baseline stigma worry or stigma change. Mean stigma change was −0.897 among participants aged 18–25 years and −0.686 among those older than 25 years, *p* = 0.217. Baseline stigma was similar by service, with mean scores of 3.47 in the Young Adult Mood Disorders Unit and 3.40 in the Adult Mood Disorders Unit, *p* = 0.601. Stigma reduction was greater in the Young Adult Unit than in the Adult Unit, although this exploratory comparison did not reach statistical significance (−1.03 vs. −0.42, p = 0.052). Length of stay did not differ significantly between services: the Young Adult Mood Disorders Unit had a mean stay of 19.0 days (*SD* = 13.5; median 14; IQR = 10–24), compared with 20.6 days (*SD* = 13.5; median = 18; IQR = 11–26) in the Adult Mood Disorders Unit, Wilcoxon rank-sum *z* = −1.084, *p* = 0.278.

Baseline depressive symptom severity was modestly associated with baseline stigma worry. The admission PHQ-9 score correlated with the admission Item 8 score, Spearman ρ = +0.262, *p* = 0.024. Admission GAD-7 score showed a non-significant trend, ρ = +0.203, *p* = 0.076. Baseline symptom severity was not associated with stigma change.

### 3.6 Independence of stigma change from clinical improvement

Stigma change was not significantly associated with clinical symptom improvement in any analysis. Continuous correlations between change scores were non-significant for PHQ-9, ρ = +0.312, *p* = 0.068, *n* = 35; GAD-7, ρ = +0.122, *p* = 0.468; and PMQ-9, ρ = −0.165, *p* = 0.375.

Comparing participants who did and did not achieve clinical thresholds yielded the same conclusion. Participants who met the PHQ-9 MCID showed a mean stigma change of −0.88 versus −0.36 among those who did not, *p* = 0.205. Participants who met the GAD-7 MCID showed a mean stigma change of −0.94 versus −0.62 among those who did not, *p* = 0.309. PHQ-9 remission was also not associated with significantly different stigma change, −0.88 versus −0.63, *p* = 0.579. No change in any other attitude item was significantly correlated with stigma change, Spearman ρ range −0.185 to +0.040, all *p* > 0.14.

### 3.7 Sensitivity analyses

Sensitivity analyses compared participants with paired admission-discharge attitude data (*n* = 64) with those without paired data (*n* = 58). The groups did not differ significantly on most characteristics, including gender (*p* = 0.181), prior hospitalization (*p* = 0.250), bipolar diagnosis (*p* = 0.102), education (*p* = 0.177), admission PHQ-9 score (mean = 16.1 vs. 14.9, *p* = 0.439), or length of stay (18.8 vs. 20.8 days, *p* = 0.820). Major depressive disorder was modestly more prevalent in the paired group (65.6% vs. 50.9%, *p* = 0.034). Item-level missingness was minimal: Item 2 had *n* = 63, Item 7 had *n* = 62, and all other paired items had *n* = 64.

## 4 Discussion

This study aimed to examine changes in attitudes toward psychiatric hospitalization from admission to discharge among adults treated in a specialty mood disorders unit, with a primary focus on hospitalization-related stigma worry, and to explore whether changes in attitudes were related to clinical improvement and length of stay. This prospective study found that psychiatric hospitalization at a specialty mood disorders unit was associated with a 26.5-percentage-point reduction in stigma worry alongside broad improvements across eight of ten paired attitude items and high satisfaction rates. These findings are consistent with modified labeling theory’s prediction that direct, positive contact with inpatient psychiatric care can revise anticipated stigma downward when the experienced reality diverges favorably from prior expectations (7). The observed decrease in stigma worry suggests that the feared social meaning of admission may be less fixed than patients expect at admission. The finding that 80.0% would return and 81.5% would recommend hospitalization on the unit after a mean stay of nearly three weeks supports the interpretation that the hospitalization experience was acceptable and meaningful for many participants.

The specificity of the length-of-stay-stigma relationship is among the most clinically and policy-relevant findings of this report. Longer hospitalization predicted greater stigma reduction, yet length of stay was not associated with change in the other nine attitude items. This pattern is consistent with therapeutic milieu models that identify sustained exposure to structured peer interaction, normalization of the hospitalization experience, staff-patient relationships, psychoeducation, and diagnostic clarification as active mechanisms of destigmatization, processes that require sufficient time to unfold (9, 10). National policy trends toward shorter inpatient psychiatric stays may therefore carry a hidden cost: attenuation of destigmatizing milieu effects that are distinct from, and not reducible to, symptomatic improvement.

The most clinically important finding was the convergence of two independent results: participants entering with the highest stigma burden showed the greatest reductions, and this change was not associated with achieving clinically meaningful improvement in depression or anxiety. Across both continuous correlations and threshold-based MCID comparisons, stigma change and clinical improvement were not significantly associated. This suggests that destigmatization may not be merely a byproduct of symptom relief but rather an active, parallel process occurring within the inpatient milieu. If stigma reduction is not driven by symptom relief, clinicians should not assume that stigma concerns resolve automatically when symptoms improve. Instead, stigma may require direct attention as its own treatment target. Practically, this supports systematic admission-time stigma screening to identify high-burden patients for targeted destigmatization programming.

The larger reductions observed among participants with high baseline stigma worry, however, should be interpreted cautiously. Regression to the mean, mathematical coupling (because baseline scores contributed to both group assignment and change scores), and floor effects may all have inflated the apparent group differences in change. Although the association between longer length of stay and greater stigma reduction is not readily explained by these statistical phenomena alone, the uncontrolled design cannot distinguish a true treatment or milieu effect from these alternative explanations. Accordingly, the baseline-severity finding should be considered hypothesis-generating and warrants replication using designs that minimize these biases. Likewise, the absence of significant associations with symptom change does not itself establish that the two processes are independent, given the small paired clinical subsamples.

Exploratory subgroup findings should be interpreted cautiously. Age group was not significantly associated with stigma change, but the young adult mood disorders unit showed a larger reduction than the adult mood disorders unit, although the difference was borderline and underpowered. One possible explanation is that young adults may experience stigma and identity-related concerns differently, particularly in relation to peer evaluation, social comparison, and emerging identity development during this life stage, which may shape how they engage with and respond to the inpatient peer environment (16). Admission PHQ-9 score was modestly associated with baseline stigma worry, suggesting that participants with more severe depressive symptoms carried greater stigma concern into hospitalization, although baseline symptom severity did not predict stigma change.

Several limitations warrant acknowledgment. This was a single-site study from a well-resourced academic tertiary care center; generalizability to community or general inpatient settings is uncertain. This was an interim analysis of an ongoing study, and the paired analytic sample included 64 of 122 enrolled participants, raising the possibility of attrition or response bias and limiting precision for subgroup analyses. Although sensitivity analyses showed no significant differences between participants with and without paired data on measured characteristics, including length of stay and admission symptom severity, participants who left the unit early or responded less well to treatment may be underrepresented among questionnaire completers, which would tend to inflate apparent attitude improvement. The Attitudes Questionnaire was study-specific rather than a validated stigma instrument such as the Internalized Stigma of Mental Illness scale; its psychometric properties are not established (5). In particular, the primary stigma outcome was a single Likert-type item, which precludes estimation of measurement reliability and has unknown construct validity. Social desirability may also have shaped discharge responses, as questionnaires completed at the end of a treatment episode can elicit favorable reporting. Major depressive disorder was modestly more prevalent in the paired group, which may introduce minor selection bias. The PHQ-9 and GAD-7 paired subsamples limited the power of independence analyses, and nonsignificant associations in these subsamples should not be interpreted as establishing independence. Finally, although length of stay was associated with stigma reduction, causality cannot be inferred from this observational design. These limitations point to clear directions for future research, which should incorporate validated stigma worry instruments, examine mechanisms of change, include comparison conditions that can separate milieu effects from regression to the mean, characterize reasons for attrition, compare young adult and adult inpatient services with adequate power, and evaluate whether stigma worry reduction persists after discharge.

Despite these limitations, this study provides prospective, paired evidence that hospitalization-related stigma can change during inpatient mood disorder treatment. The results support viewing stigma as a dynamic treatment-relevant outcome rather than a static background characteristic. They also suggest that specialty inpatient care may have destigmatizing effects that are not captured by symptom scales alone.

## 5 Conclusion

Psychiatric hospitalization at a specialty mood disorders unit was associated with a significant and clinically meaningful reduction in stigma worry and broad improvements in attitudes toward inpatient care. The magnitude of destigmatization was greatest among participants with the highest baseline stigma, and longer length of stay was associated with greater reduction in stigma worry; stigma worry change was not significantly associated with clinical symptom improvement. These findings position the inpatient therapeutic milieu as an active site of stigma intervention and provide a rationale for systematic admission-time stigma screening, targeted psychoeducation for high-stigma patients, and evidence-based advocacy for adequate inpatient length of stay in specialty mood disorders settings.

Clinicians, inpatient programs, and policymakers should act on these findings: assessing stigma worry at admission, addressing it directly as a treatment target during hospitalization, and protecting the inpatient time required for destigmatization to occur can help ensure that patients leave the hospital not only symptomatically improved but also less burdened by the fear of being known to have needed psychiatric care.

## Data Availability

The datasets presented in this article are not readily available because the data contains protected health information. Requests to access the datasets should be directed to Sarah Collica, scollic2@jhmi.edu.
